# Neurodevelopmental outcomes at 3–12 months corrected age in predominantly enteral-fed very low birth weight infants in a resource-limited South African setting

**DOI:** 10.3389/fped.2026.1888414

**Published:** 2026-07-01

**Authors:** Muneerah Satardien, J. I. Van Zyl, Lizelle van Wyk, Evette Van Niekerk, Mirjam M Van Weissenbruch

**Affiliations:** 1 Department of Paediatrics and Child Health, Faculty of Medicine and Health Sciences, Stellenbosch University, Cape Town, South Africa; 2 Division of Human Nutrition, Department of Global Health, Faculty of Medicine and Health Sciences, Stellenbosch University, Cape Town, South Africa; 3 Division of Intensive Care Neonatology, Emma Children’s Hospital, Amsterdam UMC, Vrije Universiteit, Amsterdam, Netherlands

**Keywords:** growth, neurodevelopment, premature, resource-limited, very-low-birthweight

## Abstract

**Background:**

Very low birth weight infants (VLBWIs) are at risk of neurodevelopmental impairment (NDI). Evidence linking early nutrition and growth to neurodevelopment is largely derived from high-income settings where parenteral nutrition is routine. Data from resource-limited settings using predominantly enteral feeding strategies are limited.

**Objective:**

To describe neurodevelopmental outcomes and post-discharge growth trajectories up to 12 months corrected age and identify associated factors in a predominantly enteral-fed cohort of VLBWIs.

**Methods:**

A retrospective cohort study was conducted at Tygerberg Hospital, South Africa (2021–2022). VLBWIs who received predominantly enteral nutrition and attended follow-up were included. Neurodevelopment was assessed using the Hammersmith Infant Neurological Examination (HINE) at 3, 6, and 12 months corrected age (CA). NDI was defined using age-specific HINE thresholds. Associations between clinical variables and NDI were evaluated using Fisher's exact test, Wilcoxon rank-sum tests, and logistic regression.

**Results:**

Of 296 infants with follow-up data, 190 (64.2%) attended the 12-month assessment. Most infants had HINE scores within the normal range (mean ± SD: 75.2 ± 7.7), with 5/190 (2.6%) classified as abnormal. Extrauterine growth restriction was common (33.7%) but was not associated with NDI at 12 months. Late-onset sepsis and metabolic bone disease were associated with increased odds of abnormal neurodevelopmental outcome in unadjusted analyses. In multivariable analysis, these associations persisted, although estimates were imprecise.

**Conclusion:**

In this predominantly enteral-fed cohort of VLBWIs, early motor outcomes assessed using the HINE at 12 months corrected age were reassuring despite a high prevalence of EUGR at discharge. Neonatal morbidities, particularly late-onset sepsis and metabolic bone disease, appeared more strongly associated with adverse early neurological outcomes than growth parameters alone. Longer-term follow-up is needed to determine whether later-emerging neurodevelopmental impairments become apparent beyond infancy.

## Introduction

Improving neurodevelopmental outcomes in very low birth weight infants (VLBWIs) remains a central goal of neonatal care. While optimal nutrition and growth are traditionally emphasised, the relative contributions of growth and neonatal morbidity to long-term neurodevelopment remain uncertain.

In most high-income settings, parenteral nutrition and aggressive nutritional strategies are commonly used to minimise extrauterine growth restriction (EUGR) (1, 2). However, in many low- and middle-income (LMIC) settings, resource constraints necessitate reliance on predominantly enteral feeding approaches (3, 4). Although growth outcomes in this context have been described, the implications of such strategies for both growth and neurodevelopmental outcomes remain limited (5–7). EUGR is frequently used as a surrogate marker of nutritional adequacy (8–10), yet its relationship with neurodevelopment is inconsistent. It remains unclear whether EUGR directly contributes to adverse neurodevelopmental outcomes or reflects underlying illness severity (1, 11, 12).

Comprehensive neurodevelopmental assessments, such as the Bayley Scales of Infant and Toddler Development (BSID) and Griffiths Scales of Child Development (GSCD), are considered the reference standard for neurocognitive and neurodevelopmental screening tools, but are time-consuming and require specialised training (13). The Hammersmith Infant Neurological Examination (HINE) is recommended in the International Clinical Practice Early Diagnosis of Cerebral Palsy Guidelines, particularly for resource-limited settings where comprehensive neurodevelopmental assessments and other predictive tools, such as general movements and MRI, are not easily accessible (14). The HINE provides a structured neurological assessment from 2 months onward, with 90% predictive accuracy for cerebral palsy (CP) when performed after 5 months, and exhibits good inter-observer reliability across all levels of clinical experience (15). HINE can also provide evidence concerning the risk of delayed cognitive performance at age 2 years (16).

This study aimed to describe neurodevelopmental outcomes using serial HINE assessments at 3-, 6-, and 12-months corrected age (CA) in a predominantly enteral-fed cohort of VLBWIs and to evaluate the relative contributions of growth, including EUGR, and neonatal morbidities to these outcomes.

## Materials and methods

### Study design and setting

A retrospective descriptive cohort included VLBWIs admitted to a tertiary neonatal unit in South Africa between January 1, 2021, and December 31, 2022. Infants who survived to discharge and attended neurodevelopmental follow-up up to 12 months were included. All infants received predominantly enteral nutrition.

The Tygerberg Neonatal Unit (TBH NNU) has 132 beds, including a 12-bed intensive care unit (NICU), and admits approximately 2,000 infants annually, of whom 750–800 are VLBWIs. The NICU at TBH is reserved for infants requiring invasive ventilation and/or surgery, and approximately 400–500 infants are admitted annually. VLBWIs requiring continuous positive airway pressure (CPAP), other forms of non-invasive respiratory support, umbilical venous catheters, or peripheral/central venous catheters with limited administration of PN are managed in the neonatal high care wards, outside the NICU, unless surgical intervention is required. The NNU has a high patient turnover, with infants being transferred to step-down units once clinically stable (defined as being off respiratory support and weighing >1,200–1,400 g).

During the study period, routine neonatal PN was not consistently available because of public-sector resource constraints. Infants, therefore, received intravenous 10% dextrose during feed advancement, with nutritional support provided predominantly through enteral feeding. PN was reserved for selected clinical indications, such as surgical necrotising enterocolitis, and was not routinely administered to VLBWIs.

All VLBWIs are scheduled for neurodevelopmental follow-up at approximately 3-, 6-, and 12 months CA, as part of routine care. At this assessment point, HINE and neurological clinical examinations are performed, including assessment of growth parameters. All evaluations are performed by a single clinician. Clinical follow-up data were retrospectively extracted after completion of the scheduled follow-up assessments.

### Study population and exclusion criteria

Eligible infants had a birth weight between 600 and 1,500 g, were admitted to TBH NNU within the first 24 h of life and attended at least one scheduled neurodevelopmental follow-up visit for HINE assessments at 3-, 6-, or 12 months. Exclusions included fatal genetic conditions (e.g., Trisomy 13 or 18); congenital or acquired gastrointestinal conditions necessitating PN (e.g., necrotising enterocolitis, gastroschisis, intestinal atresia, omphalocele); congenital cyanotic heart disease; and infants admitted for less than 28 days.

All data were extracted from digitised medical records. Collected data included routinely collected clinical variables and included: (1) maternal and neonatal demographic variables; (2) nutritional intake data, including daily energy and macronutrient intake (calculated using enteral feeding volumes and breast milk composition estimates) (17); (3) weight gain velocity (WGV) calculated using a 2-point exponential model (18); (4) anthropometric measurements expressed as *Z*-scores using Intergrowth-21st (IG-21ST) (for in-hospital growth) and WHO standards (for follow-up growth), as appropriate, and (5) common neonatal morbidities (sepsis, NEC, intraventricular haemorrhage, retinopathy of prematurity, bronchopulmonary dysplasia, metabolic bone disease of prematurity) (19). Transfer to step-down care (clinically stable and decision made by treating clinician) or discharge home (typically at >1,800g and full oral feeds) was treated as a single endpoint.

Neurodevelopmental impairment (NDI) was defined as an abnormal HINE score at the relevant age-specific timepoint: ≤56, ≤59, and ≤65 at 3-, 6-, and 12 months, respectively (15, 16, 20). Scores of <40 at 3-, 6-, or 12 months were indicative of CP (21). At clinical examination, CP was defined by the presence of one or more of the following clinical features: spastic paresis or dyskinetic movements, spastic diplegia accompanied by hypotonia, and sensory impairments, specifically deficits in auditory or visual modalities (22).

EUGR at discharge or transfer was defined as a decline in weight-for-age *Z*-score (WAZ) greater than 1.28 from birth to discharge/transfer based on the Intergrowth-21st (IG-21ST) charts. For this study, underweight for age (UWFA) at 12 months CA was defined as weight-for-age <10th centile on WHO growth standards. Low macronutrient intake during hospital admission was defined as intakes below ESPGHAN recommendations (10). Necrotising enterocolitis (NEC) was defined as Bell's stage 2 or more; bronchopulmonary dysplasia (BPD) was defined as the continued need for respiratory support at 36 weeks postmenstrual age (PMA); anaemia was defined as the need for red blood cell transfusion, and metabolic bone disease of prematurity (MBDP) was defined as an alkaline phosphatase as >500 IU/L and/or serum-phosphate ≤1.8 mmol/L. Retinopathy of prematurity (ROP) and intraventricular haemorrhage (IVH) were documented as the most severe scores and evaluations during hospitalisation. Hyponatremia was defined as a serum sodium level <135 mmol/L at any time during hospital admission.

### Statistical analysis

Continuous variables were summarised as means with standard deviations (SD) or medians with interquartile ranges (IQR), depending on distribution. Categorical variables were summarised as frequencies (*n*) and percentages. Group comparisons were performed using Mann–Whitney *U* tests and Fisher’s exact tests. Univariate logistic regression was used to assess associations between clinical variables and abnormal neurodevelopment. Variables demonstrating an association with the outcome at *p* < 0.10 in univariate analyses were entered into multivariable logistic regression models Odds ratios. (OR) with 95% confidence intervals (CI) were calculated using logistic regression analyses for categorical outcomes, and mean differences with 95% CIs were reported for continuous outcomes. Analyses were conducted using available data for each outcome, and no imputation of missing data was performed.

Subgroup analyses were performed for 2 groups: (1) infants still hospitalised at PMA 36 weeks and (2) EUGR infants. This allowed comparison of nutritional and growth outcomes at a standardised in-hospital time point, and evaluation of neonatal morbidities associated with neurodevelopmental impairment (NDI). A second subgroup analysis was for EUGR and non-EUGR infants. Associations were examined using unadjusted analyses.

Data were analysed using STATA 18 (StataCorp LLC, College Station, Texas, USA). Statistical significance was defined as a *p* < 0.05. Data were reported using the STROBE guidelines (23).

### Ethics approval

Ethics approval was obtained from the Health Research Ethics Committee of Stellenbosch University (N23/09/114), with a waiver of parental consent granted. Written informed consent was waived due to the retrospective nature of the study and the use of routinely collected anonymised clinical data. All research complied with the Declaration of Helsinki.

## Results

**Figure 1:**
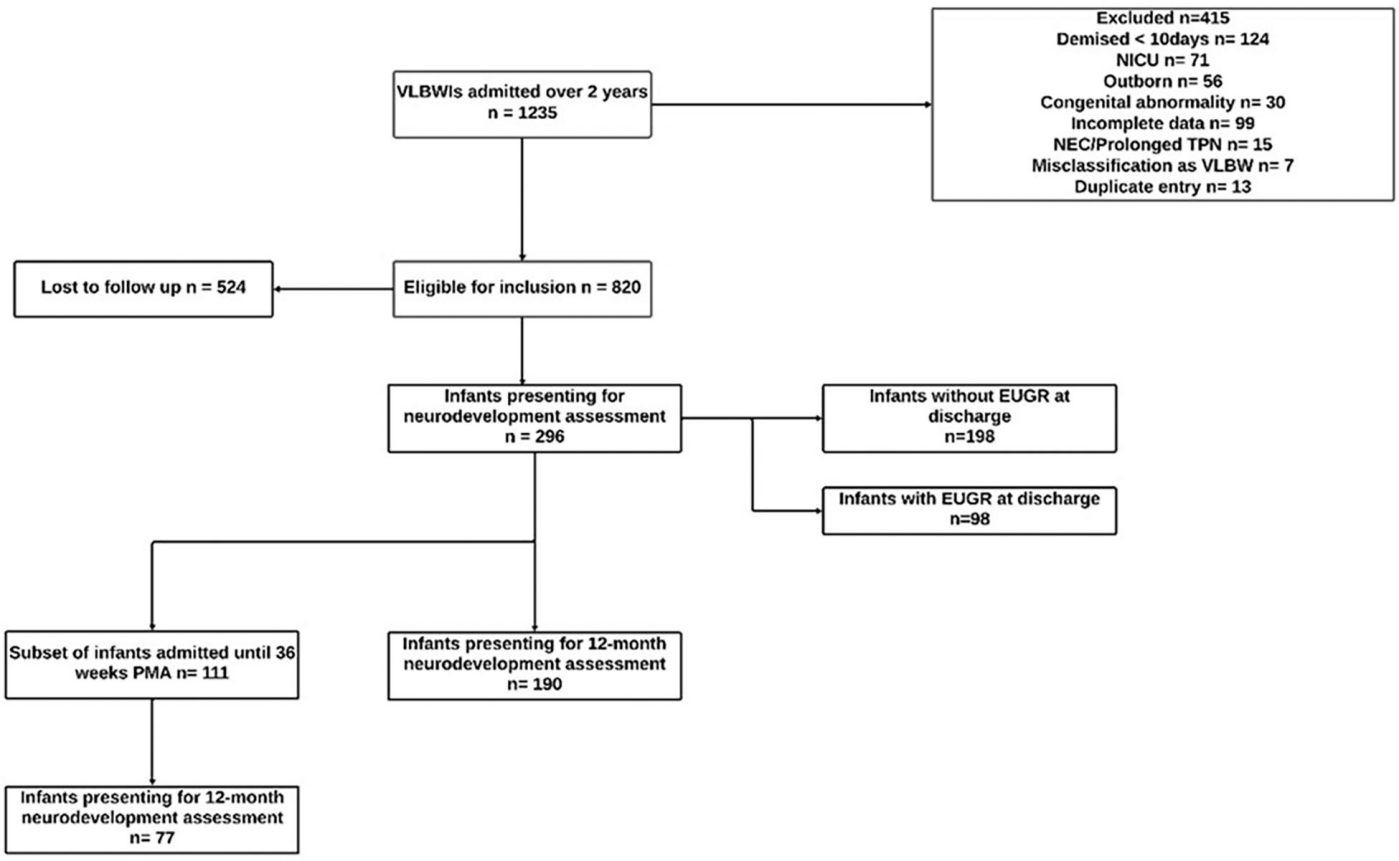
Flow diagram of study cohort and follow-up assessments. EUGR, extrauterine growth restriction; NEC, necrotising enterocolitis; NICU, neonatal intensive care unit; CA, postmenstrual age; TPN, total parenteral nutrition; VLBWIs, very low birth weight infants.

Of 820 eligible VLBWIs admitted during the study period, a total of 296 infants with available neurodevelopmental assessments and HINE scores were included in the analysis: 283/296 (95.6%) at 3 months, 115/296 (38.9%) at 6 months, and 190/296 (64.2%) at 12 months CA (Figure 1).

The mean gestational age of the infants in the cohort was 28.6 ± 1.7 weeks, and the mean birth weight was 1,044.1 ± 199.3 g (birth *Z*-score of −0.4 ± 1.6). Of the cohort, 63 infants (21.8%) had extremely low birth weight (≤1,000 g), and 42 (8.1%) were small for gestational age at birth. At discharge or transfer, 100/296 (33.7%) infants had EUGR (Supplementary Data Table S1).

To explore the potential impact of attrition bias, infants attending the 3-month HINE assessment who subsequently completed the 12-month assessment were compared with those who did not complete the 12-month evaluation. No substantial differences were observed in EUGR prevalence, neonatal morbidities, or early HINE performance between groups (Supplementary Data Table S2).

### Neurodevelopmental outcomes

Neurodevelopmental and anthropometric data at each follow-up visit are represented in Table 1.

**Table 1:** Neurodevelopmental assessment outcomes and anthropometric data.

Variable	3-Month ND follow-up	6-Month ND follow-up	12-Month ND follow-up
Total population, *n* (%)	283 (95.6)	115 (38.9)	190 (64.2)
CA, months, mean ± SD	3.1 ± 1.0	6.8 ± 3.4	12.6 ± 3.5
HINE score, mean ± SD	56.2 ± 15.0	68.0 ± 17.6	75.2 ± 7.7
Abnormal HINE score for age, *n* (%)	55.0 (19.4)	12.0 (10.4)	5.0 (2.6)
Abnormal neurological exam, *n* (%)	11.0 (3.8)	3.0 (2.6)	5.0 (2.6)
CP, *n* (%)	8.0 (2.8)	3.0 (2.6)	3.0 (1.6)
Anthropometry			
Weight, kg, mean ± SD	5.3 ± 3.2	6.9 ± 1.2	7.7 ± 2.8
WHO WAZ, mean ± SD	−2.4 ± 2.9	−1.3 ± 2.2	−0.5 ± 1.4
WHO weight <10 percentile, *n* (%)	177.0 (62.5)	61.0 (53.0)	48 (30.2)
Length, cm, mean ± SD	51.5 ± 15.0	58.7 ± 17.4	63.6 ± 12.8
WHO LAZ, mean ± SD	−1.8 ± 1.5	−1.1 ± 1.6	−1.0 ± 1.3
HC, cm, mean ± SD	31.2 ± 4.4	40.9 ± 9.5	45.0 ± 4.6
WHO HCAZ, mean ± SD	0.2 ± 1.6	0.3 ± 1.4	0.1 ± 1.4

CA, corrected age; CP, cerebral palsy; HAZ, head circumference for age *Z*-score; HC, head circumference; HINE, Hammersmith infant neurological examination; LAZ, length for age-*Z*-score; ND, neurodevelopment; WHO, World Health Organisation; WAZ, weight-for-age *Z*-score.

There was a gradual decrease over time in abnormal HINE scores, and mean HINE scores increased with age, as expected. At 3 months CA, less than one-fifth of infants met abnormal age-specific HINE score thresholds (≤56). At 6 months CA, just more than 10% of infants met abnormal age-specific HINE score thresholds (≤59), and at 12 months, less than 3% met abnormal age-specific HINE score thresholds (≤65) (Table 1). The proportion of infants diagnosed with CP remained low across all timepoints (less than 3%).

Most demographic and neonatal variables were not associated with abnormal neurodevelopmental outcomes at 3, 6, or 12 months CA (Table 2).

**Table 2:** Non-adjusted odds ratios for adverse outcome of neurodevelopmental impairment (NDI).

Variable	NDI-3		NDI-6		NDI-12		Cerebral palsy	
	*n* = 55		*n* = 12		*n* = 5		*n* = 10	
	*n* (%)/mean SD	OR (95% CI) *p*-value	*n* (%)/mean SD	OR (95% CI) *p*-value	*n* (%)/mean SD	OR (95% CI) *p*-value	*n* (%)/mean SD	CP OR (95% CI) *p*-value
Maternal variables								
Maternal HIV	5 (9.1)	0.833 (0.391–1.774) 0.636	2 (16.7)	1.952 (0.536–7.109) 0.310	0 (0.0)		2 (20.0)	1.058 (0.114–9.777) 0.460
Diabetes	1 (1.8)		1 (8.3)	9.272 (0.541–18.827) 0.124	0 (0.0)		2 (20.0)	2.150 (0.254–18.183) 0.482
Hypertensive disorder	12 (21.8)	0.625 (0.345–1.132) 0.122	6 (50.0)	5.333 (1.106–25.388) 0.159	2 (40.0)	1.058 (0.114–9.777) 0.960	1 (10.0)	1.302 (0.359–4.715) 0.687
Neonatal variables								
Male	25 (45.5)	1.189 (0.658–2.147) 0.863	9 (75.0)	3.306 (0.846–12.915) 0.085	3 (60.0)	2.485 (0.404–15.256) 0.326	6 (60.0)	1.987 (0.549–7.196) 0.295
GA	28.4 ± 2.2	0.947 (0.798–1.124) 0.863	28.5 ± 3.0	1.030 (0.769–1.380) 0.839	28.2 ± 1.6	0.930 (0.565–1.531) 0.777	28.9 ± 2.3	1.105 (0.782–1.564) 0.569
BW	1,027.7 ± 196.7	0.946 (0.813–1.100) 0.472	1,107.7 ± 229.6	1.211 (0.918–1.598) 0.176	1,055 ± 201.8	1.072 (0.696–1.651) 0.752	976.5 ± 222.3	0.824 (0.582–1.167) 0.276
SGA	10 (18.2)	1.021 (0.510–2.046) 0.952	3 (25.0)	0.956 (0.237–3.859) 0.951	1 (20.0)	0.779 (0.847–7.059) 0.825	3 (30.0)	3.318 (0.932–11.812) 0.604
ELBW	14 (25.5)	1.090 (0.604–1.965) 0.733	3 (25.0)	0.511 (0.152–1.721) 0.279	1 (20.0)	0.244 (0.026–2.230) 0.212	0 (0.0)	
Neonatal morbidities								
EUGR at discharge or transfer	14 (25.5)	1.597 (0.870–2.932) 0.131	5 (41.7)	0.544 (0.136–2.173) 0.389	1 (20.0)	2.714 (0.441–16.676) 0.281	0 (0.0)	
BPD	8 (14.5)	1.669 (0.696–4.000) 0.250	1 (8.3)	0.752 (0.088–6.395) 0.794	1 (20.0)	1.857 (0.198–17.412) 0.583	0 (0.0)	
NEC	2 (3.6)	0.679 (0.148–3.127) 0.620	0 (0.0)		1 (20.0)	4.694 (0.474–46.431) 0.186	0 (0.0)	
EOS	1 (1.8)	1.674 (0.896–3.129) 0.538	0 (0.0)		0 (0.0)		0 (0.0)	
LOS	15 (27.3)	1.674 (0.896–3.129) 0.106	1 (8.3)	0.592 (0.121–2.881) 0.516	5 (100.0)	1.510 (1.127–3.005) 0.036	1 (10.0)	1.809 (0.497–6.587) 0.368
ROP ≥ Stage II	17 (30.9)	1.211 (0.139–10.578) 0.863	4 (33.3)	0.481 (0.158–1.460) 0.197	0 (0.0)		0 (0.00)	
IVH ≥ Grade 3	2 (3.6)	1.000 (0.140–7.099) 0.367	1 (8.3)	1.270 (0.643–2.508) 0.491	0 (0.0)		6 (60.0)	1.666 (0.882–3.148) 0.116
MBDP	19 (34.5)	1.486 (0.804–2.744) 0.205	6 (50.0)	1.109 (0.328–3.746) 0.867	3 (60.0)	1.227 (1.088–5.637) 0.031	1 (10.0)	9.785 (1.222–78.337) 0.814
Anaemia requiring RBCT	24 (43.6)	1.125 (0.613–2.062) 0.704	3 (25.0)	0.982 (0.248–3.875) 0.979	2 (40.0)	0.755 (0.104–5.486) 0.782	1 (10)	8.059 (0.994–65.308) 0.051
Hyponatremia	25 (45.4)	2.31 (1.196–4.494) 0.013	5 (41.7)	0.796 (0.236–2.690) 0.715	0 (0.0)		2 (20.0)	2.836 (0.591–13.599) 0.192

BPD, bronchopulmonary dysplasia; CP, cerebral palsy; EBM, expressed breast milk; ELBW, extremely low birth weight; ELGAN, extremely low gestational age neonate <28 weeks; EUGR, extra-uterine growth restriction; EOS, early onset sepsis; IVH, intraventricular haemorrhage; LOS, late onset sepsis; MBDP, metabolic bone disease of prematurity; *n* reflects number with available exposure and outcome data for each model, ND, neurodevelopment; NEC, necrotising enterocolitis; RBCT, red blood cell transfusion; ROP, retinopathy of prematurity; SGA, small for gestational age; WAZ, weight-for-age *Z*-score; WGV, weight growth velocity.

At 3 months, only hyponatremia was associated with NDI (OR 2.32, 95% CI 1.20–4.49; *p* = 0.013). At 6 months, no variables were associated with NDI. At 12 months, late-onset sepsis (OR 1.5, 95% CI 1.12–3.00; *p* = 0.036) and metabolic bone disease (OR 1.2, 95% CI 1.08–5.60; *p* = 0.031) were associated with NDI. Anaemia requiring a red blood cell transfusion (RBCT) trended towards significance with NDI at 12 months (OR 8.05, 95% CI 0.99–65.30, *p* = 0.051). No associations with CP were present (Table 2). In multivariable analysis, late-onset sepsis and metabolic bone disease remained associated with increased odds of abnormal neurodevelopment.

### In-hospital nutrition, morbidities, and neurodevelopmental outcomes

No consistent associations were observed between low macronutrient intake at day 28 or day 56 and NDI at 3, 6, 12 months, or CP (Table 3). At 3 months, time to full feeds was associated with NDI (OR 1.49, 95% CI 1.02–1.28, *p* = 0.018).

**Table 3:** In-Hospital nutrition and associated neurodevelopment outcomes.

Variable	NDI-3		NDI-6		NDI-12		Cerebral palsy	
	*n* = 55		*n* = 12		*n* = 5		*n* = 10	
	*n* (%)/median (IQR)	OR (95% CI) *p*-value	*n* (%)/median (IQR)	OR (95% CI) *p*-value	*n* (%)/median (IQR)	OR (95% CI) *p*-value	*n* (%)/median (IQR)	OR (95% CI) *p*-value
Nutrition								
Low protein D28^a^	16 (29.1)	0.843 3 (0.352–2.018) 0.702	11 (91.7)	1.49 (0.729–3.026) 0.275	2 (40.0)	0.72 (0.325–1.604) 0.438	5 (50.0)	0.624 (0.364–1.069) 0.099
Low fat D28	3 (5.4)	3.46 (0.749–16.029) 0.130	0 (0.0)		1 (20.0)	0.285 (0.902–11.834) 0.118	1 (10.0)	4.75 (0.517–43.771) 0.240
Low carbohydrate D28	23 (41.8)	1.32 (0.704–2.479) 0.385	5 (41.7)	0.92 (0.263–3.220) 0.899	2 (40.0)	0.771 (0.125–4.742) 0.778	6 (60.0)	2.08 (0.575–7.569) 0.257
Low energy D28	8 (14.5)	1.255 (0.554–2.843) 0.585	0 (0.0)		1 (20.0)	1.269 (0.136–11.795) 0.834	3 (30.0)	2.702 (0.668–10.931) 0.163
Low protein D56	4 (7.2)	2.80 (0.672–11.669) 0.157	0 (0.0)		1 (20.0)	10.8 (0.582–20.107) 0.137	2 (20.0)	5.888 (0.814–42.582) 0.079
Low fat D56	3 (5.4)	3.64 (0.216–16.386) 0.383	0 (0.0)		1 (20.0)	0.885 (0.192–17.457) 0.303	1 (10.0)	19.5 (1.022–37.193) 0.074
Low carbohydrate D56	6 (10.9)	3.00 (0.896–10.036) 0.081	0 (0.0)		1 (20.0)	4.36 (0.252–7.530) 0.327	2 (20.0)	3.38 (0.513–22.302) 0.228
Low energy D56	3 (5.4)	5.20 (0.803–33.736) 0.084	1 (8.3)	1.01 (0.985–1.045) 0.239	1 (20.0)	28.50 (1.271–638.891) 0.035	3 (30.0)	16.88 (2.009–141.963) 0.029
Feeding and growth variables								
Time to first feed, days, median, IQR	3 (2–4)	1.066 (0.981–1.157) 0.130	4 (2–5)	1.031 (0.924–1.152) 0.583	4 (3–4)	1.054 (0.874–1.270) 0.584	2.5 (1–4)	1.096 (0.997–1.206) 0.057
Time to full feeds, days, median, IQR	8 (7–10)	1.149 (1.025–1.289) 0.018	9 (7–12)	1.031 (0.941–1.130) 0.510	9 (8–11)	1.040 (0.916–1.182) 0.544	8 (7–11)	1.141 (1.008–1.291) 0.009
Prolonged donor EBM^b^	11 (20.0)	0.461 (0.175–1.214) 0.515	3 (25.0)	2.315 (0.079–6.939) 0.530	3 (60.0)	1.196 (0.099–3.316) 0.092	0 (0.0)	
Poor WGV^c^ at D28	17 (30.9)	3.460 (0.199–6.602) 0.419	4 (33.3)	1.225 (0.056–2.274) 0.937	4 (80.0)	1.040 (0.014–2.108) 0.382	2 (20.0)	1.019 (0.014–1.739) 0.258
Poor WGV^c^ at D56	13 (23.6)	1.417 (0.415–4.834) 0.578	8 (66.7)	1.913 (0.191–19.198) 0.581	0 (0.0)		5 (50.0)	1.721 (0.190–15.590) 0.629

First feed defined as receiving 50% of prescribed feeds; full feeds defined as 150 mL/kg/day. *n* reflects number with available exposure and outcome data for each model. Estimates with wide confidence intervals reflect small, exposed groups and limited event numbers.

a Low-below ESPGHAN recommendations.

b Donor EBM for longer than 30 days.

c In-hospital weight gain <15 g/kg/day.

CP was associated with low energy provision at day 56 (OR 16.88, 95th CI 2.009–141.963, *p* = 0.029) and time to full feeds (OR 1.14, 95% CI 1.00–1.29, *p* = 0.009) (Supplementary Data Table S3).

### VLBWIs remaining hospitalised at 36 weeks PMA

Subgroup analysis was conducted on 111 infants who remained hospitalised at 36 weeks PMA (mean GA 29.9 ± 1.0 weeks, mean BW 1,057.5 ± 203.8 g). The mean change in *Z* score was −0.7, representing a decline from −1.1 ± 1.0 at birth to −1.8 ± 1.0 at 36 weeks PMA.

For the 36-week PMA subgroup, the mean HINE scores at 3, 6 and 12 months were 54.9 ± 16.7, 65.0 ± 20.7, and 75.2 ± 5.6, respectively. There were no statistical differences with the main cohort (*p* = 0.458, *p* = 0.737, *p* = 1.00, respectively) (Supplementary Figure S1). Within the 36-week PMA subgroup, NDI was observed in 25.0% (27/108) at 3 months, 15.4% (8/52) at 6 months, and 3.9% (3/77) at 12 months, while CP was diagnosed in 8.1% (9/111).

In univariate analysis, no maternal or neonatal demographic variable, neonatal morbidity, or nutritional variable was significantly associated with NDI (Table 4) at 12 months CA. Similarly, no consistent associations were observed between macronutrient intake and neurodevelopmental impairment within this subgroup (Table 4).

**Table 4:** Comparison of EUGR and non-EUGR infants.

Variable	Non-EUGR *n* = 198 (66.9)	EUGR *n* = 98 (33.1)	OR/Mean difference (95% CI)	*p*-value
	*n* (%)/mean ± SD	*n* (%)/mean ± SD		
Maternal				
Human immunodeficiency virus	50 (23.04)	11 (14.28)	0.557 (0.273–1.135)	0.107
Diabetes	7 (3.24)	8 (10.39)	3.462 (1.211–9.895)	0.020
Hypertensive disorder	116 (53.70)	41 (53.94)	1.010 (0.598–1.706)	0.971
Neonatal				
Male	118 (54.4)	48 (62.3)	1.388 (0.815–2.365)	0.227
Gestational age	28.6 ± 1.7	28.3 ± 1.8	0.900 (0.776–1.044)	0.161
Birth weight	1,047.9 ± 196.3	1,039.4 ± 208.2	1.000 (0.998–1.001)	0.747
Small for Gestational age	52 (24.07)	17 (22.07)	0.894 (0.480–1.665)	0.723
Extremely low birth weight	99 (45.62)	40 (51.94)	1.289 (0.765–2.169)	0.340
Absolute weight loss, g, median (IQR)	10.018 (7.454–14.965)	9.001 (5.596–13.161)	−1.027 (−4.532 to 2.579)	0.046
In hospital weight growth velocity, g/kg/day, mean ± SD	10.305 ± 3.579	15.042 ± 8.240	4.742 (3.033–6.414)	0.000
Discharge weight *Z*-score, median (IQR)	−2.391 (−3.454 to 1.500)	0.136 (−1.291 to 2.469)	2.531 (1.272–3.859)	0.000
Δ weight *Z*-score, birth to discharge/transfer, median (IQR)	−2.080 (−2.632 to 1.581)	0 (−0.417 to 2.469)	2.038 (1.140 to 3.376)	0.000
Neurodevelopment				
Abnormal HINE (≤ 56) at 3 months	35 (16.99)	19 (25.33)	1.658 (0.879–3.127)	0.119
Abnormal HINE (≤ 59) at 6 months	10 (12.65)	2 (5.71)	0.418 (0.087–2.018)	0.278
Abnormal HINE (≤ 65) at 12 months	3 (2.25)	1 (2.04)	0.903 (0.092–8.890)	0.930
CP	5 (2.30)	4 (5.19)	2.323 (0.607–8.885)	0.218
Nutrition and growth				
Low protein day 28^a^	55 (27.77)	29 (39.12)	1.676 (0.956–2.936)	0.073
Low fat D28	4 (2.02)	2 (2.71)	1.347 (0.242–7.515)	0.738
Low carbohydrate D28	78 (39.33)	37 (50.00)	1.538 (0.899–2.633)	0.116
Low energy D28	23 (11.61)	15 (20.27)	1.934 (0.947–3.951)	0.070
Low protein D56	4 (7.23)	4 (14.22)	2.125 (0.489–9.227)	0.314
Low fat D56	0 (0.00)	1 (3.53)	2.125 (0.489–9.227)	0.337
Low carbohydrate D56	8 (14.53)	6 (21.41)	1.602 (0.496–5.180)	0.431
Low energy D56	1 (1.81)	3 (10.71)	6.480 (0.642–65.433)	0.113
Prolonged donor expressed breast milk^b^	9 (12.13)	5 (17.27)	1.505 (0.458–4.942)	0.501
Poor weight growth velocity at D28	111 (55.55)	47 (63.52)	1.396 (0.806–2.418)	0.234
Poor weight growth velocity at D56	37 (64.91)	23 (76.6)	1.776 (0.650–4.856)	0.263
Morbidities				
Bronchopulmonary dysplasia	15 (6.91)	13 (17.11)	2.779 (1.255–6.152)	0.012
Necrotising enterocolitis	13 (5.99)	1 (1.30)	0.206 (0.027–1.605)	0.132
Early-onset sepsis	21 (10.6)	17 (17.3)	1.385 (0.998–2.335)	0.394
Late-onset sepsis	59 (27.11)	21 (27.22)	1.004 (0.560–1.801)	0.059
EUGR at transfer/discharge	198 (66.89)	98 (33.10)	2.71 (0.442–16.683)	0.281
Retinopathy of prematurity ≥ Stage II	6 (4.41)	2 (8.33)	1.970 (0.373–10.389)	0.448
Intraventricular haemorrhage ≥ Grade 3	6 (3.36)	0 (0.00)	1.62 (0.670–3.745)	0.889
Metabolic bone disease of prematurity	106 (54.08)	28 (36.84)	0.495 (0.287–0.853)	0.089
Anaemia requiring RBCT	99 (47.37)	44 (61.97)	1.811 (1.044–3.141)	0.035
Hyponatremia	121 (56.54)	51 (66.23)	1.508 (0.875–2.598)	0.139

HINE, Hammersmith infant neurological examination.

a Low-below ESPGHAN recommendations.

b Donor EBM for longer than 30 days.

c In-hospital weight gain <15 g/kg/day.

Low fat at day 28 was associated with NDI at 3 months (OR 12.00, 95% CI 1.27–112.66, *p* = 0.030), and low fat at day 56 was associated with NDI at 12 months (OR 45.00, 95th CI 1.49–1,358.27, *p* = 0.029). Low energy on day 56 was associated with CP (OR 12.444, 95th CI 1.474–105.048, *p* = 0.02, Supplementary Data Table S4).

### EUGR infants

**Figure 2:**
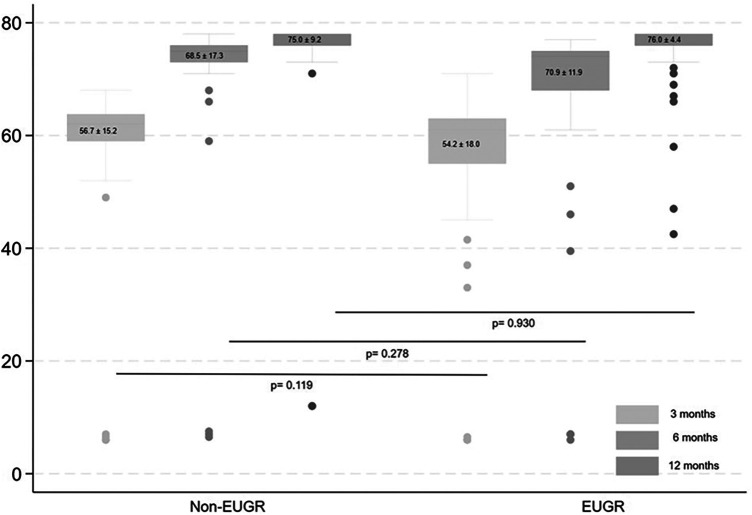
HINE scores at 3, 6, and 12 months corrected age stratified by EUGR status.

Subgroup analysis was conducted on 98 infants (33.1%) who were EUGR at discharge/transfer. The mean HINE scores at 3, 6 and 12 months were 54.2 ± 18.0, 70.9 ± 11.9, and 76.0 ± 4.4, respectively, for the EUGR infants. Neurodevelopmental outcomes at follow-up were comparable between groups, with no consistent association between EUGR status and adverse HINE scores (Figure 2).

Demographic characteristics were similar between groups, except for a higher prevalence of maternal diabetes in the EUGR infants. Neurodevelopmental outcomes did not differ. In contrast, EUGR infants had increased rates of BPD, LOS, MBDP, and anaemia requiring transfusion (Table 4).

EUGR infants also demonstrated poorer early nutrition and growth trajectories, with greater weight loss, slower weight gain velocity, and lower discharge weights compared to non-EUGR infants. Despite similar timing of feed initiation, these deficits persisted throughout hospitalisation (Supplementary Data Tables S5, S6).

Multivariate regression was performed on variables with a *p*-value <0.1, where LOS and MBDP were associated with increased odds of abnormal neurodevelopmental outcome at 12 months in infants with EUGR (Supplementary Data Tables 7).

## Discussion

In this cohort of very low birth weight (VLBW) infants followed to 12 months CA in a resource-limited setting, the overall burden of neurodevelopmental impairment (NDI) as assessed by the Hammersmith Infant Neurological Examination (HINE) was low, with only 2.6% of infants demonstrating abnormal HINE scores at 12 months and 3.4% diagnosed with cerebral palsy (CP). Mean HINE scores increased progressively across follow-up visits, reflecting expected neurological maturation during infancy. Despite the high prevalence of EUGR at discharge (33.1%) and evidence of suboptimal early growth, meaningful somatic recovery was observed after discharge, particularly in linear growth and head growth. In this cohort, EUGR was not associated with adverse early motor outcomes assessed by the HINE at 12 months corrected age among infants available for follow-up. However, this finding should be interpreted cautiously, as previous studies have reported associations between postnatal growth restriction and adverse neurodevelopmental outcomes in preterm populations.

The HINE captures tone, posture, reflexes, and motor responses that evolve rapidly during the first year of life as cortical and corticospinal pathways mature (14, 16). Early abnormalities in preterm infants are therefore common and often transient (15). In this cohort, nearly one-fifth of infants had abnormal age-specific HINE scores at 3 months, yet this proportion declined markedly by 12 months. This trajectory likely reflects the gradual resolution of immature motor patterns and tone abnormalities that are frequently observed in preterm infants during early infancy. Previous longitudinal studies have similarly shown that early neurological examinations may overestimate long-term impairment, while persistent abnormalities after 9–12 months are more predictive of later motor disability (15, 24).

The marked decline in abnormal findings over time supports the use of serial neurological assessments rather than single early evaluations (14, 15, 25). At the same time, outcomes at 12 months should be interpreted as reflecting early motor integrity rather than definitive long-term neurodevelopment.

The relatively low prevalence of CP (3.4%) compared with reports from high-income settings (5%–10%) should be interpreted cautiously (26). Survival bias and loss to follow-up may have excluded more vulnerable infants, while assessment at 12 months may underestimate later-emerging motor, cognitive, and behavioural impairments. Some infants classified as normal in early infancy are subsequently diagnosed with CP or other motor disorders later in childhood (27). Thus, the low prevalence of abnormal HINE scores and CP at 12 months is reassuring for early motor outcomes among the assessed survivors but does not rule out later neurodevelopmental impairment (28, 29). Nonetheless, repeated neurological assessments strengthened early detection of significant motor dysfunction.

Previous studies have demonstrated associations between postnatal growth restriction and adverse neurodevelopmental outcomes in preterm populations, particularly with respect to later cognitive, behavioural, and motor functioning (30–33). However, the effects of early nutritional deficits may be subtle and not readily detectable during early infancy. The lack of consistent associations between EUGR, early macronutrient intake, and neurodevelopmental outcomes in this study should therefore be interpreted with caution. Nutritional exposures are closely confounded by illness severity, and early motor assessments may not capture the full impact of nutritional deficits. Similarly, variables such as time to full enteral feeding may reflect overall clinical instability rather than isolated nutritional effects. Differences in EUGR definitions, follow-up duration, neurodevelopmental assessments, and study populations may also contribute to discrepancies between studies.

Most maternal and neonatal demographic variables were not significantly associated with NDI across follow-up timepoints (34). Gestational age and birth weight, although important determinants of survival and morbidity in preterm infants, may have had limited variability within this cohort, thereby reducing their statistical association with early neurodevelopmental outcomes. The relatively low prevalence of severe neurological injury in this cohort may therefore partly explain the absence of strong demographic predictors of impairment.

The association between hyponatremia and NDI at 3 months, which was not sustained at later follow-up, likely reflects transient effects rather than persistent injury, as it may influence early neurological examination through altered neuronal excitability or osmotic shifts, and so serves as a marker of systemic instability and illness severity in this cohort. The absence of association at later timepoints supports a limited direct causal role in long-term motor impairment (35).

Late-onset sepsis (LOS) and metabolic bone disease of prematurity (MBDP) demonstrated associations with neurodevelopmental impairment at 12 months, although event numbers were small. The association between LOS and adverse neurodevelopmental outcomes is well described in the literature and is believed to result from systemic inflammatory responses during critical periods of brain development (36, 37). Pro-inflammatory cytokines released during sepsis may contribute to white matter injury, impaired oligodendrocyte maturation, and disrupted neural connectivity (38).

Similarly, MBDP may reflect prolonged nutritional deficiencies or chronic illness (39–41), which may adversely affect neurodevelopment through mechanisms involving impaired mineral metabolism, reduced physical activity, and overall illness burden. MBDP may be best understood here as an indicator of chronic neonatal adversity, not merely a skeletal complication. The same logic may apply to the near-significant signal for anaemia requiring transfusion: transfusion exposure may represent more severe illness, repeated phlebotomy loss, inflammation, or prolonged respiratory support rather than an isolated hematologic mechanism.

The subgroup of infants remaining hospitalised at 36 weeks PMA represents a particularly vulnerable population with greater illness severity and slower postnatal growth trajectories (42). Within this subgroup, early neurodevelopmental impairment rates were higher, although associations with nutritional variables were inconsistent and estimates were imprecise. The exploratory nature of these analyses and the limited number of outcome events warrant cautious interpretation, but they suggest that prolonged hospitalisation and associated morbidities may identify infants at higher developmental risk.

Weight-for-age improved progressively during follow-up, with reductions in the proportion of infants below the 10th percentile over time. Similarly, WHO length-for-age *Z*-scores improved between 3- and 12-month's CA, suggesting meaningful post-discharge linear catch-up growth despite substantial early postnatal growth restriction. Head circumference-for-age *Z*-scores remained relatively preserved throughout follow-up, with mean values approximating population norms at all assessment points. This preservation of head growth, despite early somatic growth deficits, may reflect relative brain sparing and could partly explain the normal early neurological outcomes observed in this cohort.

This apparent dissociation between somatic growth and early neurological outcomes has been described previously and may also reflect differences in developmental domains and timing. While early neurological examinations primarily assess motor and tone-related functions, the effects of early nutritional deficits may manifest later in cognitive and behavioural outcomes (32, 43). The absence of association in this study should therefore not be interpreted as evidence that nutritional deficits have no long-term neurodevelopmental consequences. Furthermore, comparisons with cohorts receiving routine PN should be interpreted cautiously, as nutritional exposures and healthcare resources differ substantially across settings.

Follow-up patterns represent an important finding, with high attendance at 3 months, a marked decline at 6 months, and partial recovery at 12 months, likely reflecting structural constraints typical of resource-limited settings. Barriers such as transport costs, caregiver employment, and competing priorities likely contributed to attrition. This has important methodological implications, as differential follow-up may bias outcome estimates and attenuate observed associations. Clinically, it is also significant, as higher-risk infants are often least likely to attend, underscoring the challenge of sustained developmental surveillance in constrained health systems. Although some loss to follow-up occurred, infants attending the 3-month HINE assessment who subsequently completed the 12-month evaluation demonstrated similar EUGR prevalence and early neurological performance to those who did not complete the 12-month assessment, reducing concern regarding substantial attrition bias.

Several limitations should be acknowledged. Loss to follow-up remains an important limitation, as attrition may introduce selection bias and underestimate impairment rates. Outcomes were assessed only up to 12 months CA using the HINE, precluding evaluation of later-emerging cognitive, language, executive, and behavioural outcomes. Although the HINE is a validated tool for the early identification of neurological dysfunction and cerebral palsy, it primarily assesses motor and neurological function during infancy. Consequently, the findings of this study should be interpreted as reflecting early motor neurological outcomes rather than overall long-term neurodevelopmental status.

Nutritional exposures were estimated using standard nutrient composition values rather than individualized human milk analysis. Consequently, day-to-day variations in human milk composition were not accounted for, introducing the potential for exposure misclassification and measurement error that may have attenuated associations between nutritional intake and neurodevelopmental outcomes. Therefore, the absence of significant associations between early macronutrient intake and early motor outcomes should be interpreted cautiously and should not be considered evidence of a lack of biological effect.

The relatively small number of adverse outcomes particularly the limited number of abnormal HINE assessments at 12 months corrected age, reduced statistical power and resulted in wide confidence intervals in some analyses. Consequently, multivariable models may have been underpowered and susceptible to overfitting, limiting the precision and stability of adjusted effect estimates. Both positive and negative associations, particularly those relating to EUGR and nutritional exposures, should therefore be interpreted cautiously. Additionally, residual confounding related to illness severity, socioeconomic factors, and post-discharge care may have influenced the observed associations.

In conclusion, VLBW infants in this resource-limited setting demonstrated reassuring early motor outcomes at 12 months corrected age despite a high prevalence of EUGR at discharge. Longitudinal follow-up showed evidence of post-discharge catch-up growth, particularly in linear and head growth parameters. Neonatal morbidities, particularly LOS and MBDP, were more strongly associated with adverse outcomes, highlighting the importance of optimising overall neonatal care and preventing systemic illness alongside nutritional support. Strengthening infection prevention, metabolic monitoring, consistent enteral feeding practices, and accessible long-term neurodevelopmental follow-up may therefore have the greatest impact on outcomes in resource-limited settings. Further research is needed to evaluate later neurocognitive outcomes to better understand the complex interactions between neonatal illness, nutrition, post-discharge growth recovery, and long-term development.

## Data Availability

The raw data supporting the conclusions of this article will be made available by the authors, without undue reservation.
