# Supplementary material for: Neurodevelopmental outcomes at 3–12 months corrected age in predominantly enteral-fed very low birth weight infants in a resource-limited South African setting
Source: Front Pediatr. 2026 Jul 1;14:1888414. doi: 10.3389/fped.2026.1888414 (PMC13369299; doi:10.3389/fped.2026.1888414)

**Supplementary data**

Supplementary Table 1- Population demographics for the cohort

| Variable |  |
| --- | --- |
| **Maternal Variables** | |
| Maternal age (years), mean ± SD | 28.3 ± 6.5 |
| Gravidity, mean ± SD | 2.6 ±1.4 |
| Parity, mean ± SD | 1.5± 1.3 |
| Antenatal care, n (%) | 284 (95.9) |
| Maternal HIV positive, n (%) | 107 (36.4) |
| HIV treated, n (%) | 107 (100) |
| Maternal syphilis, n (%) | 28 (9.5) |
| Maternal hypertensive disorder, n (%) | 126 (42.6) |
| Maternal diabetes, n (%) | 23 (7.7) |
| **Neonatal variables** | |
| Gestational age (weeks), mean ± SD | 28.6 ± 1.7 |
| Gestational age < 32 weeks, n (%) | 100 (33.8) |
| Gestational age < 28 weeks, n (%) | 84 (28.4) |
| Birth weight (g), mean ± SD | 1044.1 ± 199.3 |
| Extremely low birth weight, n (%) | 63 (21.8) |
| Birth weight Z-score, mean ± SD | -0.4 ± 1.6 |
| Birth weight centile, mean ± SD | 43.6 ± 29.2 |
| Birth length (cm), mean ± SD | 37.0 ± 3.8 |
| Birth head circumference (cm), mean ± SD | 27.0 ± 1.8 |
| Male, n (%) | 129 (43.6) |
| Neonatal HIV, n (%) | 8 (5.9) |
| Small for gestational age, n (%) | 42 (8.1) |
| Apgar at 1 minute, median (IQR) | 7.0 (6.0-8.0) |
| Apgar at 5 minutes, median (IQR) | 8.0 (8.0-9.0) |
| Apgar at 10 minutes, median (IQR) | 9.0 (8.5-10.0) |

HIV- Human Immunodeficiency Virus, SD- Standard deviation, IQR- Interquartile range

Supplementary Table 2: Characteristics of infants attending the 3-month HINE assessment who did and did not complete the 12-month HINE assessment

| **Variable** | **3-month and 12-month attendees (n = 190)** | **Lost after 3 months (n = 106)** | **p-value** |
| --- | --- | --- | --- |
| Gestational age (weeks), mean ± SD | 28.4 ± 1.8 | 28.8 ± 1.8 | 0.045 |
| Birth weight (g), mean ± SD | 1026.9 ± 201.9 | 1072.8 ± 192.7 | 0.054 |
| Male sex, n (%) | 113 (59.4) | 54 (50.9) | 0.019 |
| EUGR at discharge, n (%) | 49 (26.8 | 28 (25.0) | 0.736 |
| LOS, n (%) | 54 (28.4) | 27 (25.4) | 0.293 |
| NEC, n (%) | 10 (66.7) | 5 (33.3) | 0.790 |
| BPD, n (%) | 22 (75.9) | 7 (24.1) | 0.098 |
| IVH grade III/IV, n (%) | 3 (2.2) | 3 (3.8) | 0.672 |
| Abnormal HINE at 3 months, n (%) | 30 (17.0) | 20 (18.9) | 0.748 |
| HINE score at 3 months, mean ± SD | 56.6 ± 15.4 | 55.6 ± 16.6 | 0.603 |

Supplementary Table 3: Non-adjusted odds ratios for adverse Neurodevelopmental Outcome for infants ≥36 weeks

|  | **NDI-3: n = 30 (27.8)** | | | **NDI-6: n = 8 (15.4)** | | | **NDI-12: n = 3 (3.9)** | | | **Cerebral Palsy: n = 9 (8.1)** | | |
| --- | --- | --- | --- | --- | --- | --- | --- | --- | --- | --- | --- | --- |
| **Variable** | **n (%)/**  **mean ±SD** | **OR (95% CI)** | **p-value** | **n (%)/**  **mean ± SD** | **OR (95% CI)** | **p-value** | **n (%)/**  **mean ±SD** | **OR (95% CI)** | **p-value** | **n (%)/**  **mean ± SD** | **OR (95% CI)** | **p-value** |
| **Maternal variables** | | | | | | | | | | | | |
| HIV | 2 (7.4) | 0.718  (0.239-2.157) | 0.555 | 1 (12.5) | 1.296  (0.223-7.535) | 0.773 | 0 (0.0) |  |  | 1 (11.1) | 2.184  (0.501-9.526) | 0.298 |
| Diabetes | 0 (0.0) |  |  | 1 (12.5) | 6.143  (0.343-10.937) | 0.217 | 0 (0.0) |  |  | 1 (11.1) | 1.696  (0.185-15.558) | 0.640 |
| Hypertensive disorder | 11 (40.7) | 0.464  (0.196-1.094) | 0.079 | 6 (75.0) | 3.286  (0.597-18.097) | 0.172 | 2 (66.7) | 2.353  (0.204-27.093) | 0.493 | 5 (55.5) | 1.027  (0.261-4.046) | 0.970 |
| **Neonatal variables** | | | | | | | | | | | | |
| Male | 12 (44.4) | 0.739  (0.314-1.737) | 0.488 | 6 (75.0) | 5.250  (0.946-29.147) | 0.058 | 2 (66.7) | 3.286  (0.285-37.924) | 0.340 | 5 (55.5) | 1.583  (0.402-6.241) | 0.511 |
| GA | 29.1  ± 2.0 | 1.102  (0.894-1.360) | 0.363 | 29.1 ± 2.3 | 1.108  (0.793-1.547) | 0.547 | 28.8 ± 1.9 | 0.963  (0.515-1.799) | 0.905 | 29.1 ± 2.0 | 1.034  (0.736-1.454) | 0.846 |
| BW | 1000.8  ± 201.2 | 1.152  (0.935-1.419) | 0.183 | 979.4  ± 210.6 | 1.211  (0.855-1.713) | 0.281 | 971.0  ± 191.8 | 1.117  (0.626-1.993) | 0.707 | 1000.9  ± 200.9 | 0.942  (0.663-1.337) | 0.737 |
| SGA | 8 (29.6) | 0.554  (0.230-1.335) | 0.188 | 3 (37.5) | 0.789  (0.167-3.722) | 0.765 | 1 (33.3) | 0.694  (0.060-7.993) | 0.769 | 5 (55.5) | 1.715  (0.435-6.765) | 0.441 |
| ELBW | 12 (44.4) | 0.577  (0.247-1.348) | 0.204 | 3 (37.5) | 0.280  (0.059-1.340) | 0.111 | 1 (33.3) | 0.287  (0.025-3.318) | 0.318 | 6 (66.7) | 1.579  (0.374-6.664) | 0.534 |
| EUGR at discharge or transfer | 12 (44.4) | 0.892  (0.382-2.084) | 0.792 | 3 (37.5) | 0.720  (0.153-3.391) | 0.678 | 2 (66.7) | 2.111  (0.183-24.302) | 0.549 | 4 (44.4) | 0.974  (0.247-3.838) | 0.970 |
| **Neonatal Morbidities** | | | | | | | | | | | | |
| BPD | 7 (25.9) | 1.790  (0.655-4.891) | 0.256 | 1 (12.5) | 0.625  (0.067-5.822) | 0.680 | 1 (33.3) | 1.781  (0.152-20.926) | 0.646 | 2 (22.2) | 1.233  (0.237-6.413) | 0.803 |
| NEC | 2 (7.4) | 0.625  (0.125-3.128) | 0.567 | 0 (0.0) |  |  | 1 (33.3) | 6.900  (0.530-89.826) | 0.140 | 1 (11.1) | 1.292  (0.145-11.525) | 0.819 |
| EOS | 1.(14.3) | 0.414  (0.048-3.590) | 0.423 | 0 (0.0) |  |  | 0 (0.0) |  |  | 0 (0.0) |  |  |
| LOS | 13 (26.5) | 1.294  (0.556-3.010) | 0.549 | 1 (12.5) | 0.206  (0.023-1.825) | 0.156 | 1 (33.3) | 0.557  (0.048-6.414) | 0.639 | 4 (44.4) | 0.974  (0.247-3.838) | 0.970 |
| ROP ≥ Stage II | 14 (23.3) | 0.609  (0.159-2.327) | 0.479 | 4 (50.0) | 1.003  (0.094-3.664) | 0.254 | 2 (66.7) | 0.419  (0.034-5.129) | 0.459 | 5 (55.5) | 0.491  (0.084-2.862) | 0.599 |
| IVH ≥ Grade 3 | 2. (50.0) | 2.522  (0.335-18.983) | 0.369 | 1 (12.5) | 2.917  (0.227-37.425) | 0.411 | 0 (0.0) |  |  | 1 (11.1) | 3.167  (0.294-34.131) | 0.342 |
| Metabolic bone disease of prematurity | 18 (28.6) | 1.217  (0.508-2.914) | 0.659 | 6 (75.0) | 1.667  (0.298-9.310) | 0.561 | 3 (100.0) | 4.538  (0.226-11.083) | 0.286 | 8 (88.9) | 6.143  (0.740-50.998) | 0.093 |
| Anaemia requiring blood transfusion | 9 (20.5) | 0.754  (0.294-1.936) | 0.557 | 3 (37.5) | 0.581  (0.086-3.918) | 0.577 | 1 (33.3) | 2.284  (0.089-8.413) | 0.192 | 4 (44.4) | 2.942  (0.346-24.998) | 0.323 |
| Hyponatremia | 23 (26.7) | 1.311  (0.434-3.967) | 0.631 | 5 (62.5) | 0.370  (0.073-1.878) | 0.230 | 3 (100.0) | 1.140  (0.055-3.683) | 0.568 | 7 (77.8) | 0.864  (0.167-4.481) | 0.862 |

Supplementary Table 4: In-Hospital Nutrition and associated Neurodevelopment outcomes for ≥36 weeks CA subgroup

|  | **NDI-3: n = 30 (27.8)** | | | **NDI-6: n = 8 (15.4)** | | | **NDI-12: n = 3 (3.9)** | | | **CP: n = 9 (8.1)** | | |
| --- | --- | --- | --- | --- | --- | --- | --- | --- | --- | --- | --- | --- |
| **Variable** | **n(%) /median (IQR)** | **OR (95% CI)** | **p-value** | **n(%) /median (IQR)** | **OR (95% CI)** | **p-value** | **n(%) /median (IQR)** | **OR (95% CI)** | **p-value** | **n(%) /median (IQR)** | **OR (95% CI)** | **p-value** |
| **Nutrition** | | | | | | | | | | | | |
| *Low Protein D28 | 13 (48.1) | 0.843  (0.352–2.019) | 0.702 | 2 (25.0) | 0.275  (0.050–1.525) | 0.140 | 2 (66.7) | 1.684  (0.146–19.441) | 0.676 | 5 (55.6) | 1.332  (0.337–5.266) | 0.683 |
| Low Protein D56 | 4 (14.8) | 3.500  (0.760–16.118) | 0.108 | 0 (0.0) |  |  | 1 (33.3) | 8.200  (0.441–152.521) | 0.158 | 2 (22.2) | 5.889  (0.814–42.582) | 0.079 |
| Low Fat D28 | 4 (14.8) | 12.000  (1.278–112.661) | 0.030 | 0 (0.0) |  |  | 1 (33.3) | 11.167  (0.778–160.375) | 0.076 | 1 (11.1) | 2.844  (0.283–28.576) | 0.375 |
| Low Fat D56 | 1 (3.7) | 3.000  (0.177–50.976) | 0.447 | 0 (0.0) |  |  | 1 (33.3) | 45.000  (1.491–1358.273) | 0.029 | 1.0 (11.1) | 14.500  (0.758–277.366) | 0.076 |
| Low Carbohydrate D28 | 9 (33.3) | 0.783  (0.322–1.899) | 0.588 | 1 (12.5) | 0.500  (0.109–2.303) | 0.374 | 1.(33.3) | 1.043  (0.090–12.100) | 0.973 | 3 (33.3) | 1.276  (0.300–5.417) | 0.741 |
| Low Carbohydrate D56 | 3.(11.1) | 3.343  (0.917–12.180) | 0.067 | 0.(0.0) |  |  | 1 (33.3) | 3.600  (0.206–62.797) | 0.380 | 2.(22.2) | 2.909  (0.433–19.555) | 0.272 |
| Low Energy D28 | 9 (33.3) | 1.097  (0.448-2.685) | 0.822 | 1.(12.5) | 0.286  (0.032–2.556) | 0.262 | 1 (33.3) | 1.342  (0.115–15.671) | 0.814 | 3.(33.3) | 1.400  (0.325–6.024) | 0.651 |
| Low Energy D56 | 3 (11.1) | 3.382  (0.542-9.079) | 0.326 | 0.(0.0) |  |  | 1 (33.3) | 22.000  (0.978–494.760) | 0.052 | 2 (22.2) | 12.444  (1.474–105.048) | 0.021 |
| **Feeding and Growth Variables** | | | | | | | | | | | | |
| Time to first feed, days, median, IQR | 4 (14.8) | 1.791  (0.463-7.048) | 0.465 | 1 (12.5) | 1.537  (0.185-5.519) | 0.557 | 1 (33.3) | 4.871  (0.398-9.139) | 0.279 | 1 (11.1) | 1.521  (0.182-12.888) | 0.521 |
| Time to full feeds, days, median, IQR | 1 (44.4) | 1.373  (0.591-3.188) | 0.536 | 6 (75.0) | 0.862  (0.193-3.951) | 0.251 | 3 (100.0) | 2.486  (0.126-3.925) | 0.573 | 7 (77.8) | 0.966  (0.247-3.826) | 0.255 |
| **Prolonged donor EBM | 9 (33.3) | 1.16  (0.48–2.79) | 0.745 | 6 (75.0) | 1.16  (0.244–5.533) | 0.853 | 3 (100.0) | 2.487  (0.189-2.792) | 0.735 | 7 (77.8) | 1.53  (0.39–6.07) | 0.544 |
| Poor WGV^#^ at D28 | 3 (2.0-3.0) | 2.08  (0.411–10.593) | 0.377 | 3 (2.0-4.0) | 0.994  (0.866-1.141) | 0.933 | 3 (2.0-4.0) | 1.047  (0.851-1.289) | 0.662 | 3 (2.0-3.0) | 1.08  (0.11–10.54) | 0.945 |
| Poor WGV^#^ at D56 | 8 (7.0-9.0) | 2.08  (0.461–10.591) | 0.377 | 8.(7.0-9.0) | 0.997  (0.884-1.124) | 0.958 | 8 (7.0-9.0) | 1.029  (0.891-1.187) | 0.701 | 8 (7.0-9.0) | 1.08  (0.11–10.54) | 0.945 |

*Low-Below ESPGHAN recommendations; **donor EBM for longer than 30 days; # in-hospital weight gain <15g/kg/day; First feed defined as receiving 50% of prescribed feeds; Full feeds defined as 150mL/kg/day. Footnote: n reflects number with available exposure and outcome data for each model. Estimates with wide confidence intervals reflect small, exposed groups and limited event numbers

Supplementary Table 5: Non-adjusted odds ratios for adverse Neurodevelopmental Outcome by EUGR status

|  | **NDI-3: n = 55** | | | **NDI-6: n = 12** | | | **NDI-12: n = 5** | | | **Cerebral Palsy: n = 2** | | |
| --- | --- | --- | --- | --- | --- | --- | --- | --- | --- | --- | --- | --- |
| **Variable** | Non-EUGR n=30 (54.6) | EUGR n=25 (45.4) | p-value | Non-EUGR n=7 (58.3) | EUGR n=5 (41.7) | p-value | Non-EUGR n= 3 (60.0) | EUGR n= 2 (40.0) | p-value | Non-EUGR n= 2 (100.00) | EUGR n=0 | p-value |
| **Maternal** | | | | | | | | | | | | |
| HIV | 5 (16.7) | 5 (20) | 0.750 | 3 (42.9) | 1 (20.0) | 0.408 | 1 (33.3) | 0 (0.0) | 0.361 | 1 (50.0) | 0 (0.0) |  |
| Diabetes | 0 (0.0) | 0 (0.0) |  | 0 (0.0) | 1 (20.0) | 0.217 | 0 (0.0) | 0 (0.0) |  | 0 (0.0) | 0 (0.0) |  |
| Hypertensive disorder | 14 (46.7) | 10 (40) | 0.620 | 6 (85.7) | 4 980.0) | 0.793 | 1 (33.3) | 2 (100.0) | 0.136 | 1 (50.0) | 0 (0.0) |  |
| **Neonatal** | | | | | | | | | | | | |
| Male | 13 (43.3) | 16 (64.0) | 0.126 | 2 (28.6) | 1 (20.0) | 0.217 | 1 (33.3) | 1 (50.0) | 0.709 | 1 (50.0) | 0 (0.0) |  |
| GA | 28.2 (28-30) | 28.9 (27-30) | 0.456 | 31 (29-31) | 28 (27-29) | 0.045 | 30 (28-30) | 30 (28-30) | 0.527 | 27.5 (27-28) |  |  |
| BW | 1015 (865-1155) | 990 (910-1090) | 0.980 | 1160 (1070-1265) | 960 (930-1430) | 0.684 | 1165 (1070-1280) | 1155 (1010-1277) | 0.968 | 1120 (1030-1210) |  |  |
| SGA | 5 (17.2) | 8 (32.0) | 0.206 | 1 (16.7) | 2 (40.0) | 0.387 | 0 (0.0) | 1 (50.0) | 0.171 | 0 (0.0) | 0 (0.0) |  |
| ELBW | 14 (46.7) | 13 (52.0) | 0.694 | 4 (57.1) | 1 (20.0) | 0.198 | 0 (0.0) | 1 (50.0) | 0.187 | 0 (0.0) | 0 (0.0) |  |
| **Morbidities** | | | | | | | | | | | | |
| BPD | 3 (10.0) | 5 (20) | 0.295 | 0 (0.0) | 1 (20.0) | 0.217 | 0 (0.0) | 1 (50.0) | 0.711 | 0 (0.0) | 0 (0.0) |  |
| NEC | 1 (3.3) | 1 (4.0) | 0.895 | 1 (100.00) | 0 (0.0) |  | 0 (0.0) | 1 (50.0) | 0.171 | 0 (0.0) | 0 (0.0) |  |
| EOS | 2 (6.7) | 1 (4.0) | 0.665 | 0 (0.0) | 0 (0.0) |  | 1 (33.3) | 0 (0.0) | 0.361 | 1 (50.0) | 0 (0.0) |  |
| LOS | 10 (33.3) | 10 (40.0) | 0.609 | 1 (14.3) | 1 (20.0) | 0.793 | 0 (0.0) | 2 (100.0) | 0.059 | 1 (50.0) | 0 (0.0) |  |
| ROP ≥ Stage II | 18 (90.0) | 14 (71.4) | 0.162 | 0 (0.0) | 1 (20.0) | 0.214 | 0 (0.0) | (0.0) | 0.171 | 2 (100.0) | 0 (0.0) |  |
| IVH ≥ Grade 3 | 2 (10.5) | 0 (0.0) | 0.146 | 0 (0.0) | 1 (20.0) | 0.549 | 0 (0.0) | 2 (100.0) | 0.046 | 2 (100.0) | 0 (0.0) |  |
| Metabolic bone disease of prematurity | 21 (75) | 9 (37.5) | 0.006 | 4 (57.12) | 3 (60.0) | 0.921 | 1 (33.3) | 2 (100.0) | 0.089 | 1 (50.0) | 0 (0.0) |  |
| Anaemia requiring blood transfusion | 15 (50.0) | 13 (59.1) | 0.516 | 4 (57.1) | 1 (50.0) | 0.858 | 1 (33.3) | 1 (50.0) | 0.248 | 0 (0.0) | 0 (0.0) |  |
| Hyponatremia | 24 (80.0) | 17 (68) | 0.309 | 3 (42.9) | 4 (80.0) | 0.198 | 3 (100.0) | 2 9100.0) | 0.548 | 2 (100.0) | 0 (0.0) |  |

BPD- bronchopulmonary dysplasia; BW- birth weight; ELBW- extremely low birth weight; EOS- early onset sepsis; EUGR- extrauterine growth restriction; GA- gestational age; HIV- Human Immunodeficiency Virus; IVH- intraventricular haemorrhage; LOS- late-onset sepsis; NDI- neurodevelopmental impairment; NEC- necrotising enterocolitis; ROP- retinopathy of prematurity; SGA- small for gestational age

Supplementary Table 6: In-Hospital Nutrition and associated ND outcomes by EUGR status

|  | **NDI-3: 55** | | | **NDI-6: 12** | | | **NDI-12: 5** | | | **Cerebral Palsy: 2** | | |
| --- | --- | --- | --- | --- | --- | --- | --- | --- | --- | --- | --- | --- |
| **Variable** | Non-EUGR | EUGR | p-value | Non-EUGR | EUGR | p-value | Non-EUGR | EUGR | p-value | Non-EUGR | EUGR | p-value |
| **Nutrition** | | | | | | | | | | | | |
| *Low Protein D28 | 7 (22.6) | 9 (40.9) | 0.225 | 1 (11.1) | 1 (33.3) | 0.682 | 1 (50.0) | 1 (33.3) | 0.700 | 0 (0.0) | 0 (0.0) |  |
| Low Protein D56 | 1 (8.3) | 3 (37.5) | 0.153 | 0 (0.0) | 0 (0.0) |  | 0 (0.0) | 1 (100.0) | 0.500 | 0 (0.0) | 0 (0.0) |  |
| Low Fat D28 | 2 (6.5) | 2 (9.1) | 0.400 | 0 (0.0) | 0 (0.0) |  | 0 (0.0) | 1 (33.3) | 0.500 | 0 (0.0) | 0 (0.0) |  |
| Low Fat D56 | 0 (0.0) | 1 (12.5) | 0.400 | 0 (0.0) | 0 (0.0) |  | 0 (0.0) | 1 (100.0) | 1.000 | 0 (0.0) | 0 (0.0) |  |
| Low Carbohydrate D28 | 11 (35.5) | 12 (54.4) | 0.121 | 4 (44.4) | 1 (33.3) | 0.700 | 1 (50.0) | 1 (33.3) | 0.520 | 0 (0.0) | 0 (0.0) |  |
| Low Carbohydrate D56 | 1 (8.3) | 4 (50.0) | 0.109 | 0 (0.0) | 0 (0.0) |  | 0 (0.0) | 0 (0.0) |  | 0 (0.0) | 0 (0.0) |  |
| Low Energy D28 | 4 (12.9) | 5 (22.7) | 0.464 | 0 (0.0) | 1 (33.3) | 0.250 | 0 (0.0) | 0 (0.0) |  | 0 (0.0) | 0 (0.0) |  |
| Low Energy D56 | 1 (8.3) | 2 925) | 0.584 | 0 (0.0) | 0 (0.0) |  | 0 (0.0) | 1 (100.0) | 0.587 | 0 (0.0) | 0 (0.0) |  |
| **Feeding and Growth Variables** | | | | | | | | | | | | |
| Time to first feed, days, median, IQR | 3 (2-.2) | 3 (2-4.5) | 0.304 | 3 (2-5) | 4 (3.5-4) | 1.000 | 4 (3.5-4.5) | 4 (3.5-4) | 1.000 | 11 (9-13.5) | 0 (0.0) |  |
| Time to full feeds, days, median, IQR | 8 (7-9) | 8 (7-10.5) | 0.299 | 9 (7-9) | 11 (10-11.5) | 0.257 | 10.5 (9.8-11.2) | 8 (8-9.5) | 0.374 | 9 (9-10) | 0 (0.0) |  |
| **Prolonged donor EBM | 2 (22.2) | 4 (44.4) | 0.620 | 1 (33.3) | 0 (0.0) | 1.000 | 1 (100.0) | 0 (0.0) | 0.333 | 1(100.0) | 0(0.0) |  |
| Poor WGV^#^ at D28 | 32 (100.0) | 23 (100.0) | 0.980 | 9 (100.0) | 3 (100.0) |  | 2 (100.0) | 3 (100.0) |  | 0 (0.0) | 0 (0.0) |  |
| Poor WGV^#^ at D56 | 12 (100.0) | 8 (100.0) | 0.978 | 3 (100.0) | 2 (100.0) |  | 0 (0.0) | 0 (0.0) |  | 0 (0.0) | 0 (0.0) |  |

*Low-Below ESPGHAN recommendations: protein <3.5 g/kg/day, fat <4.8 g/kg/day, carbohydrate <11.6 g/kg/day, energy <115 kcal/kg/day. ; **donor EBM for longer than 30 days; # in-hospital weight gain <15g/kg/day; First feed defined as receiving 50% of prescribed feeds; Full feeds defined as 150mL/kg/day. Footnote: n reflects number with available exposure and outcome data for each model. Estimates with wide confidence intervals reflect small, exposed groups and limited event numbers

Supplementary Table 7: Multivariate analysis for Potential Predictors of Abnormal ND Outcome at 12 months for EUGR subgroup

| Variable | Univariate OR (95% CI) | p-value | Multivariable OR (95% CI) | p-value |
| --- | --- | --- | --- | --- |
| **Maternal factors** | | | | |
| Maternal Diabetes | 3.4 (1.21-9.89) | 0.020 | 1.1 (0.78–3.51) | 0.890 |
| **Neonatal factors** | | | | |
| **Neonatal morbidities** | | | | |
| Bronchopulmonary Dysplasia | 2.7 (1.25–6.15) | 0. 012 | 3.9 (0.95-16.35) | 0.710 |
| Late-onset sepsis | 1.0 (0.56–1.80) | 0.059 | 1.8 (1.03-3.14) | 0.037 |
| Metabolic bone disease | 0.4 (0.28–0.85) | 0.089 | 2.2 (1.02-4.70) | 0.037 |
| Anaemia requiring blood transfusion | 1.8 (1.04–3.14) | 0.035 | 2.0 (1.12-3.89) | 0.865 |
| **Feeding and Growth Variables** | | | | |
| Low Protein Day 28 | 1.6 (0.95–2.93) | 0.073 | 1.8 (0.79–4.51) | 0.980 |
| Low Energy D28 | 1.9 (0.94–3.95) | 0.070 | 2.2 (0.88–5.60) | 0.651 |
| Absolute weight loss, g, median (IQR) | -1.0 (-4.53-2.57) | 0.046 | −1.2 (−4.0 - 2.9) | 0.370 |
| In hospital weight growth velocity, g/kg/day, mean ± SD | 4.7 (3.03 - 6.41) | 0.000 | 2.7 (1.39 - 6.85) | 0.927 |
| Discharge weight Z-score, median (IQR) | 2.5 (1.27 - 3.85) | 0.000 | 2.5 (1.41- 7.37) | 0.898 |
| Δ weight Z-score, birth to discharge/transfer, median (IQR) | 2.0 (1.14 - 3.37) | 0.000 | 3.7 (2.03 - 4.41) | 0.542 |

Supplementary Figure 1: HINE scores at 3, 6, and 12 months CA in the whole cohort and infants hospitalised at 36 weeks CA


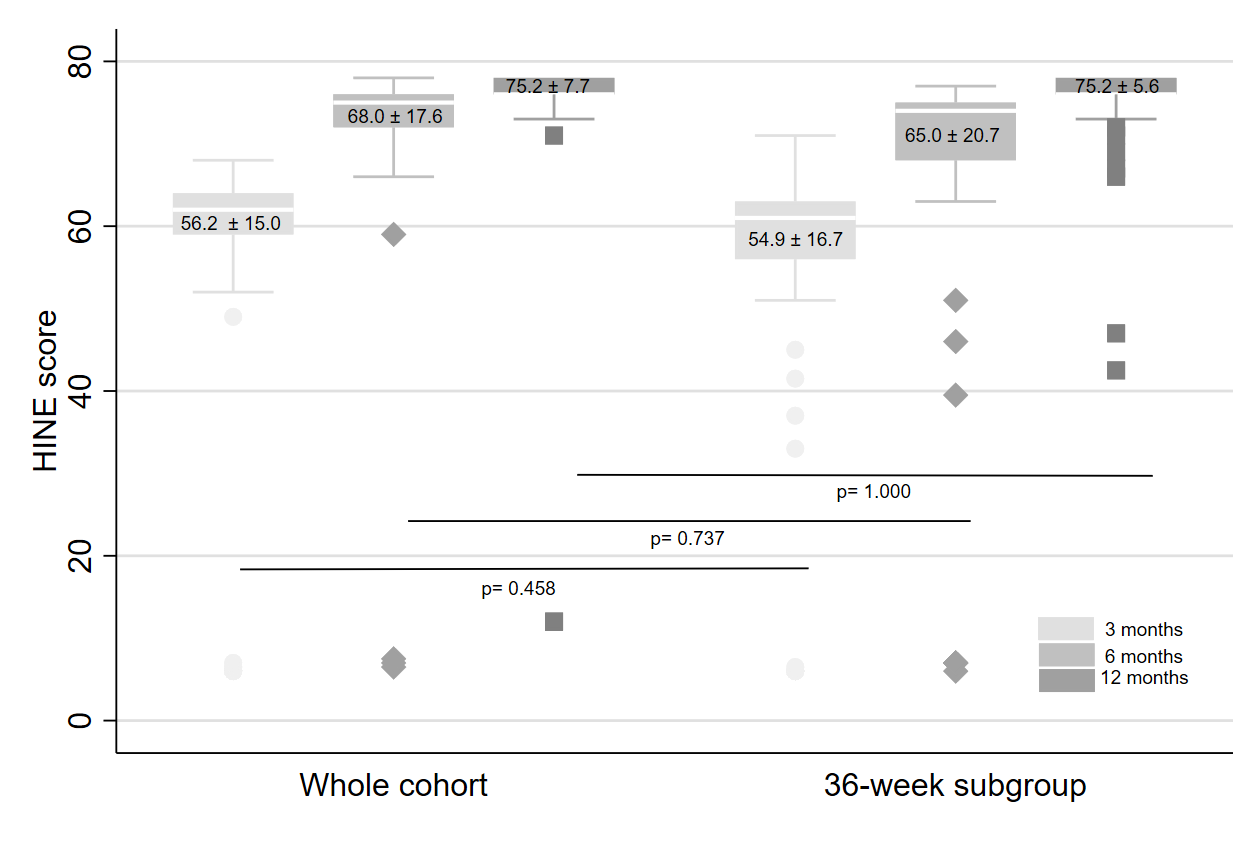

Supplement: Supplementary file 1 [file Supplementaryfile1.docx]
